# Change and HOAP for the best

**DOI:** 10.7554/eLife.64945

**Published:** 2020-12-22

**Authors:** Claudia Castillo-González, Dorothy E Shippen

**Affiliations:** Department of Biochemistry and Biophysics, Texas A&M UniversityCollege StationUnited States

**Keywords:** essential gene, positive selection, telomere, conflict, retrotransposon, domestication, *D. melanogaster*

## Abstract

HOAP is a telomere-binding protein that has a conserved role in *Drosophila*, but it also needs to evolve quickly to restrict telomeric retrotransposons.

**Related research article** Saint-Leandre B, Christopher C, Levine MT. 2020. Adaptive evolution of an essential telomere protein restricts telomeric retrotransposons. *eLife* **9**:e60987. doi: 10.7554/eLife.60987

Telomeres are specialized structures that ‘cap’ and protect the ends of linear chromosomes. They allow the cell to distinguish the ends of chromosomes from abnormal double-stranded breaks, and they protect the genetic information from being lost after replication (Barbero Barcenilla and Shippen, 2019). Losing telomeres leads to harmful anomalies, such as chromosomes fusing together: these structures are therefore conserved in all organisms with linear chromosomes, including (but not limited to) all animals, plants, insects and fungi (de Lange, 2018).

Telomeres are formed of dedicated protein complexes and sequences of repetitive DNA elements; these are tightly compacted into heterochromatin and therefore more difficult to transcribe. In *Drosophila* species, telomeres consist of a specialized type of genetic elements known as the HTT-array, which is then capped with a terminin complex that includes the proteins HOAP and HipHop (Casacuberta, 2017; Pardue and DeBaryshe, 2008). In fact, loss of terminin is lethal at the embryonic stage due to catastrophic chromosome fusions (Cenci et al., 2003; Mlotshwa et al., 2010). In addition, the DNA in *Drosophila* telomeres is wrapped around proteins decorated with a specific mark recognized by Heterochromatin Protein 1 (HP1 for short) to ensure that they remain in their compacted form. HP1 is also part of the telomere cap, as it directly interacts with HOAP and HipHop.

The HTT-array consists of ‘parasitic’ DNA sequences known as retrotransposons, which, when transcribed, can ultimately ‘copy and paste’ themselves into different regions in the genome. This ability plays a key role in lengthening and maintaining telomeres, as HTTs typically get inserted at chromosome-ends (Casacuberta, 2017), but they can also cause genome instability if these sequences replicate without control. Remarkably, when expressed, some HTT transcripts are processed into small RNAs that direct a silencing complex known as piRISC, which severs HTT transcripts, limiting their expression (Akkouche et al., 2017). In addition, piRISC guides the deposition of the genetic marks that help recruit HP1 and compact chromatin, also reducing the transcription of the retrotransposons (Sato and Siomi, 2018). In fact, loss of heterochromatin results in over-proliferation of retrotransposons and telomere deregulation (Akkouche et al., 2017; Khurana et al., 2010; Perrini et al., 2004; Radion et al., 2018).

Given the critical role of telomeres, it is intriguing that flies have evolved a protective mechanism that involves parasitic elements and factors like HOAP and HipHop, which are two of the fastest evolving proteins in *Drosophila* (Saint-Leandre and Levine, 2020). How can conserved functions be fulfilled by an ever-changing molecular machinery? Now, in eLife, Bastien Saint-Leandre, Courtney Christopher and Mia Levine, from the University of Pennsylvania, report that HOAP performs more than one function, which is why it needs to evolve fast (Saint-Leandre et al., 2020).

First, Saint-Leandre et al. analyzed the sequences of the *cav* gene, which encodes HOAP, in different *Drosophila* species. This revealed that advantageous mutations of the protein were being selected for, a hallmark of adaptive evolution. CRISPR/Cas9 technology was harnessed to generate mutant *D. melanogaster* flies with the gene from another *Drosophila* species, *D. yakuba; D. melanogaster* flies with a tagged version of the native HOAP (HOAP[mel]) served as a control. Flies expressing the *D. yakuba* version of HOAP (HOAP[yak]) were viable, confirming that the essential role of HOAP was conserved between species despite the two proteins having diverging sequences.

Next, Saint-Leandre et al. followed the phenotypes of telomeres in the engineered *D. melanogaster* flies expressing HOAP[yak] through fifty generations. This revealed that, in *D. melanogaster*, both versions of the HOAP protein protected telomere ends, but only HOAP[mel] regulated HTT expression and telomere length. The telomeres in flies carrying the *D. yakuba* variant did not have the genetic marks recognized by HP1 and recruited much less of this protein. Additionally, these insects accumulated less piRISC, despite the expression of the HTT-array being upregulated (Figure 1). This observation is consistent with previous studies showing that depleting telomeres of piRISC produces abnormal telomeric chromatin (Akkouche et al., 2017; Radion et al., 2018). Strikingly, HTT overexpression in flies expressing HOAP[yak] resulted in the array inserting itself outside telomeres, potentially disrupting essential genes. Indeed, these females produced fewer progeny over their lifetime than those expressing the native HOAP[mel]. This fitness cost is consistent with catastrophic genome damage.

**Figure 1.**
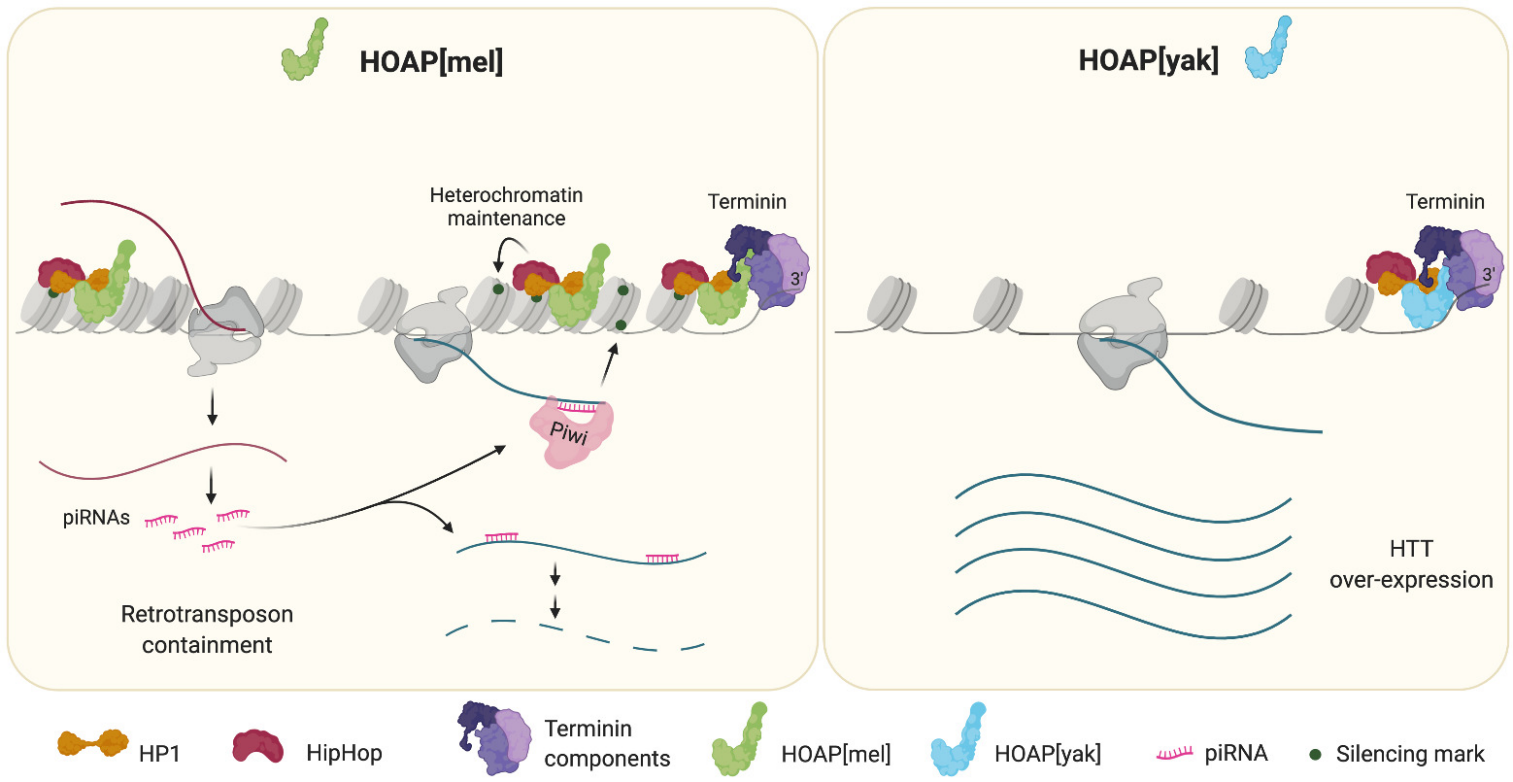
Comparison of telomere phenotypes in *Drosophila melanogaster* flies expressing HOAP from either *D. melanogaster* (HOAP[mel]) or *D. yakuba* (HOAP[yak]). Left: the telomeres of a *D. melanogaster* fly expressing HOAP[mel]. The complex formed by HOAP[mel] (green), HipHop (maroon) and HP1 (dark orange) binds the telomere. Bi-directional transcription of the HTT-array produces both full-length transcripts (dark blue lines) and shorter transcripts (bright pink lines) that bind the protein Piwi (light pink) to form piRISC. This complex drives the degradation of the full-length HTT transcripts to prevent retrotransposons from being inserted into other regions of the chromosome. Additionally, piRISC guides the deposition of silencing marks (dark green circles) which serve to recruit HP1 to the telomeres. This leads to chromatin compaction, reducing HTT-array transcription and providing a second layer of control over the retrotransposons. The complex formed by HOAP, HipHop and HP1 also binds to terminin (purple) to cap and protect the chromosome end. Right: telomeres of a *D. melanogaster* fly expressing the *cav* gene from *D. yakuba*, HOAP[yak]. While HOAP[yak] (light blue) can bind to HipHop and HP1 and bind terminin to effectively cap the chromosome ends, it fails to perform the roles of the native HOAP in regulating the HTT-array. This means that the telomeres of flies expressing HOAP[yak] have lower levels of HP1 and do not have silencing marks to aid in the compaction of chromatin. Consequently, chromatin becomes relaxed, increasing the transcription of the HTT-array. The full-length transcripts of the retrotransposons that are produced are not degraded because these telomeres do not support the accumulation piRISC. This accelerates retrotransposon replication, initially elongating the telomeres, but then allowing the elements to travel to other regions of the chromosome, potentially disrupting other genes (figure created using BioRender.com).

These results show that telomere-capping and HTT regulation are two independent functions converging in HOAP, and that while telomere-capping is essential and must be conserved, HTT regulation is not. Coupled with evidence that the *D. melanogaster* and *D. yakuba* genes have undergone adaptive evolution since their divergence, this indicates that it is HOAP’s role in regulating the HTT-array that requires fast evolution. This is potentially in response to the HTT-array constantly adapting to escape control.

The work by Saint-Leandre et al. resolves the apparent paradox of a fast-evolving protein being responsible for an essential and strictly conserved role. It also predicts that HOAP, HipHop and HP1 participate in two independent complexes for telomere function: one which involves terminin and protects the ends of the chromosomes, and a second that demands constant innovation to contain the genetic parasitic elements that form telomeres. Understanding how retrotransposons are contained by this second complex and why females are more susceptible to dysregulation of HTT are two fascinating mysteries for future investigation.
